# Quercetin Inhibits Left Ventricular Hypertrophy in Spontaneously Hypertensive Rats and Inhibits Angiotensin II-Induced H9C2 Cells Hypertrophy by Enhancing PPAR-γ Expression and Suppressing AP-1 Activity

**DOI:** 10.1371/journal.pone.0072548

**Published:** 2013-09-10

**Authors:** Lei Yan, Ji Dong Zhang, Bo Wang, Yi Jing Lv, Hong Jiang, Gui Lin Liu, Yun Qiao, Ming Ren, Xue Feng Guo

**Affiliations:** 1 Department of Traditional Chinese Medicine, Qilu Hospital Affiliated to Shandong University, Jinan, Shandong, China; 2 Laboratory of Cardiovascular Remodeling and Function Research, Qilu Hospital Affiliated to Shandong University, Chinese Ministry of Education and Chinese Ministry of Health, Jinan, Shandong, China; 3 Shandong University of Traditional Chinese Medicine, Jinan, Shandong, China; 4 The Second Hospital Affiliated to Shandong University of Traditional Chinese Medicine, Jinan, Shandong, China; The Ohio State Unversity, United States of America

## Abstract

**Background:**

Quercetin is the most abundant flavonoid in fruit and vegetables and is believed to attenuate cardiovascular disease. We hypothesized that quercetin inhibits cardiac hypertrophy by blocking AP-1 (c-fos, c-jun) and activating PPAR-γ signaling pathways.

**Methodology/Principal Findings:**

The aim of this study was to identify the mechanism underlying quercetin-mediated attenuation of cardiac hypertrophy. Quercetin therapy reduced blood pressure and markedly reduced the ratio of left ventricular to body weight (LVW/BW) (*P*<0.05, vs. spontaneously hypertensive rats (SHRs)). In vitro, quercetin also significantly attenuated Ang II-induced H9C2 cells hypertrophy, as indicated by its concentration dependent inhibitory effects on [^3^H]leucine incorporation into H9C2 cells (64% reduction) and by the reduced hypertrophic surface area in H9C2 cells compared with the Ang II group (*P*<0.01, vs. Ang II group). Concurrently, we found that PPAR-γ activity was significantly increased in the quercetin-treated group both in vivo and in vitro when analyzed using immunofluorescent or immunohistochemical assays (*P*<0.05, vs. SHRs or *P*<0.01, vs. the Ang II group). Conversely, in vivo, AP-1 (c-fos, s-jun) activation was suppressed in the quercetin-treated group, as was the downstream hypertrophy gene, including mRNA levels of ANP and BNP (*P*<0.05, vs. SHRs). Additionally, both western blotting and real time-PCR demonstrated that PPAR-γ protein and mRNA were increased in the myocardium and AP-1 protein and mRNA were significantly decreased in the quercetin-treated group (*P*<0.05, vs. SHRs). Furthermore, western blotting and real time-PCR analyses also showed that transfection with PPAR-γ siRNA significantly increased AP-1 signaling and reversed the effects of quercetin inhibition on mRNA expression levels of genes such as ANP and BNP in hypertrophic H9C2 cells.

**Conclusions:**

Our data indicate that quercetin may inhibit cardiac hypertrophy by enhancing PPAR-γ expression and by suppressing the AP-1 signaling pathway.

## Introduction

Cardiac hypertrophy results in congestive heart failure and is a major world-wide cause of sudden death. Cardiac hypertrophy is characterized by an enhanced expression of proteins and a gene profile that is reminiscent of early embryonic development in cardiomyocytes. A series of transcription factors including the immediate-early genes, such as c-fos and c-jun, which are components of AP-1, and its downstream target genes including atrial natriuretic peptide (ANP) and B-type natriuretic peptide (BNP) have been implicated in the development of cardiac hypertrophy. Moreover, hallmarks of cardiac hypertrophy, such as BNP and ANP, were significantly increased in diseased hearts [1]–[3]. Activator protein-1 contains c-fos and c-jun, which together make a heterodimer complex that plays a significant role in cardiomyocyte hypertrophy, and direct inhibition of AP-1 activity significantly decreased cardiac hypertrophy in myocardial tissue [4]–[6]. These observations have contributed to speculation that inhibition of these hypertrophic signals may be effective, patent strategies in the treatment of pathological hypertrophy and heart failure. Peroxisome proliferator-activated receptors (PPARs) are transcription factors belonging to the nuclear receptor gene family that heterodimerize with the retinoid X receptor and bind to PPAR response elements (PPREs) in target gene promoters [7]. Another study also demonstrated the PPAR-γ-dependent pathway is critical in the inhibition of cardiac hypertrophy both in vivo and vitro [8]. Furthermore, we also have shown previously that PPAR-γ regulates gene expression in a DNA-independent fashion by interfering with other signaling pathways, such as the activator protein-1 (AP-1) pathway [9], [10].

Quercetin (3,3′,4′,5,7-pentahydroxyflavone) is a member of a group of naturally occurring compounds and is one of the most widely distributed bioflavonoids [11]. The very extensive biological effects of flavonoids, including their anti-inflammatory [12], anti-oxidant [13], anti-atherosclerotic [14], and anti-hypertensive [15] properties facilitate an important role for quercetin in the prevention of cardiovascular diseases. Furthermore, it has also been revealed to show agonistic effects on peroxisome proliferator activated receptors [16] and attenuates Monocyte Chemoattractant Protein-1 gene expression in glucose-primed aortic endothelial cells via AP-1 [17].

Recently, it has been shown that quercetin robustly inhibits hypertrophy of cardiomyocytes in vivo [15], [18]. However, the molecular mechanisms by which quercetin exerts its cardioprotective effects remain largely unknown, and no studies have addressed the effects of quercetin on intracellular signaling pathways. Therefore, the purpose of this study is to elucidate the potential mechanisms underlying the cardiomyocyte protective effects of quercetin and further, to investigate the biologic target of quercetin involved in cardiac hypertrophy.

## Methods

### Cell Lines, Antibodies, and Reagents

**Figure 1:**
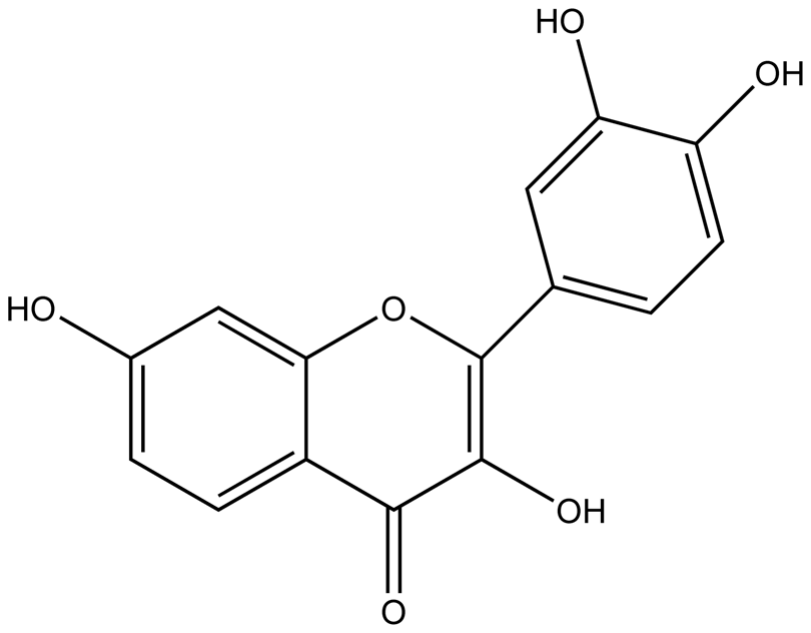
The molecular structure of quercetin.

H9C2 rat cardiomyocyte cells were obtained from the Cell Bank of Type Culture Collection of the Chinese Academy of Sciences, Shanghai, China. Quercetin (the molecular structure of quercetin is shown in Figure 1) [19] and Ang II were purchased from Sigma Aldrich (Sigma Aldrich, China). Antibodies against PPAR-γ and c-fos were obtained from Abcam (Abcam, UK). The antibody against c-jun was purchased from Cell Signaling Technology (Cell Signaling Technology, UK). Secondary immunoglobulin G conjugated antibodies and diaminobenzidine were purchased from Zhongshan Goldenbridge Biotechnology (Zhongshan Goldenbridge Biotechnology, China). TRIzol reagent was from Invitrogen (Invitrogen, Carlsbad, CA, USA). The PrimeScript™ RT reagent kit with gDNA Eraser (Perfect Real Time) and SYBR Green PCR kit were obtained from TaKaRa Corp (TaKaRa Corp., Kyoto, Japan). HRP-conjugated secondary antibodies was purchased from Bioworld (Bioworld, Biotechnology, China). The BCA protein kit was obtained from Zhongshan Goldenbridge Biotechnology (Zhongshan Goldenbridge Biotechnology, China).

### Ethics Statement

The experiments conformed to the Animal Management Rule of the Chinese Ministry of Health (documentation 55, 2001), and the experimental protocol was approved by the Animal Care and Use Committee of Shandong University.

### Animal protocol

Twenty four 8-week-old male SHRs and eight 8-week-old male Wistar–Kyoto (WKY) rats obtained from Slaccas (Shanghai, China) were used in this study. Animals were housed in a temperature-controlled (22±0.5°C) room, and rats had free access to standard rodent chow and water. A 12-h/12-h light/dark cycle was maintained. Spontaneously hypertensive rats were randomly divided into three groups: one group (SHR) was treated with vehicle (1 ml of 1% methylcellulose) and the other two groups were treated with either a low dose of quercetin (SHR+QL; 5 mg.kg^−1^, mixed in 1 ml of 1% methylcellulose) or a high dose of quercetin (SHR+QH; 10 mg.kg^−1^, mixed in 1 ml of 1% methylcellulose). Age-matched WKY rats were treated with vehicle (1 ml of 1% methylcellulosa) and used as a control group. Quercetin or vehicle was delivered by gavage at the same time once daily for 12 weeks. Systolic blood pressure was measured using a noninvasive tail-cuff system (ALC-NIBP), as previously described [20], once every week. Echocardiography was performed at the beginning and at the end of the experiment. After transthoracic echocardiography was performed, the animals were anesthetized using sodium pentobarbital and the left ventricular weight (LVW) and body weight (BW) ratio was determined. The heart was either fixed in 4% paraformaldehyde and embedded in paraffin for histopathological analysis and immunohistochemical staining or fresh myocardial tissues were stored at −80°C for real-time PCR and western blotting analysis.

### Blood pressure measurements

Systolic blood pressure was measured weekly at 18–20 h after administration of the vehicle or quercetin in conscious, prewarmed, restrained rats using tail-cuff plethysmography [20]. At least seven determinations were made during every session, and the mean of the lowest three values that varied within 5 mmHg was regarded as the systolic blood pressure value.

### Tissue preparation and morphometric analysis

After treatment, the hearts were fixed in 4% paraformaldehyde, embedded in paraffin, and sectioned at 4-µm intervals. Hematoxylin and eosin staining and Masson's trichrome staining were performed using standard procedures. The collagen volume fraction was quantified blindly using quantitative morphometry with an automated image analysis system (Image-Pro Plus, Version 7.0, Media Cybernetics, Silver Spring, MD, USA). The cardiac collagen volume fraction was calculated as a ratio of (the percentage of the sum of total interstitial collagen area) to (the sum of total connective tissue and muscle area in the entire visual field of the section) while excluding perivascular collagen, as reported previously [21]. Measurements from 3 heart sections (8–10 fields per section) per rat were averaged for all parameters.

### Transmission Electron Microscopy

Left ventricular myocardial tissues of approximately 0.5 mm×1 mm×1 mm from each group were fixed using 2% glutaraldehyde overnight and then washed three times using 0.2 mol L^−1^ phosphate buffer, re-fixed with 1% osmium tetraoxide, washed again with 0.2 mol L^−1^ phosphate buffer and dehydrated using an ethanol series. The samples were immersed in Epon812 resin acetone (1∶1) for 30 minutes and then embedded for convergence overnight at 70°C.The tissue was cut into pieces and slices at 50-nm thickness were generated using an ultra-microtome (LKB880, LKB Produkter AB, Bromma, Sweden). Embedded sections were stained using lead citrate and observed using a transmission electron microscope (TEM, H-7000FA, Hitachi, Tokyo, Japan).

### Echocardiography

The rats were anesthetized lightly using sodium pentobarbital. Transthoracic echocardiography was performed using a Philips 7500 Ultrasound System (Philips Medical Systems, Andover, MA, USA) with a 7.0-MHz transducer. Transmission gel was used between the transducer and the animal's chest to optimize the image. Images were captured from M-mode, two-dimensional (2-D), pulse wave (PW) Doppler and acoustic density. All measurements of nuclear magnetic resonance were calculated by the same observer based on the average of six consecutive cardiac cycles. At the level of the chordae tendineae, the wall thickness and left ventricular dimensions were obtained from a long-axis view. Specific dimensions, such as left ventricular end-diastolic internal diameter (LVIDd), left ventricular end-diastolic posterior wall thickness and end-diastolic interventricular septal thickness (LVPWd and IVSd, respectively), were calculated using the area-length method and the left ventricular ejection fraction (LVEF) was determined according to Teichholz's formula:
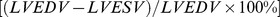
where LVEDV and LVESV represent the end-diastolic and end-systolic volumes, respectively. Mid-wall fractional shortening was calculated as follow:
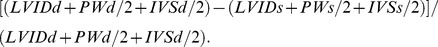
Transmitral flow velocity parameters including peak E, peak A and the E/A ratio were evaluated after pulsed Doppler. The mitral valve pulsed Doppler recordings were obtained from the apical four-chamber view [22].

### Immunohistochemical staining

The chest was opened quickly and a small piece of cardiac tissue was taken from the left ventricular apical endocardium. Formalin-fixed paraffin-embedded tissue blocks were serially sectioned at 4-µm. Sections were deparaffinized and washed in 0.01 mol/L PBS. Endogenous peroxidases were quenched using 3% hydrogen peroxide in methanol for 10 minutes. Antigen retrieval was performed by microwaving the sections for 10 minutes. The sections were then blocked using 5% normal goat serum at 37°C for 20 minutes followed by incubation at 4°C with affinity-purified rabbit monoclonal c-fos (Abcam UK, dilution 1∶100), c-jun (Cell Signaling Technology, UK, dilution 1∶200) and/or PPAR-γ (Abcam. UK, dilution 1∶50) overnight. Incubation with PBS instead of the primary antibody served as a negative control. Sections were then incubated with a secondary-conjugated immunoglobulin G antibody (Zhongshan Goldenbridge Biotechnology Co., China; dilution 1∶100). Sections were then washed three additional times with PBS for 3 minutes at room temperature. Two additional washing steps were performed using phosphate buffered saline, sections were developed using diaminobenzidine (Zhongshan Goldenbridge Biotechnology Co., China; dilution 1∶100) for 5 minutes in the dark and counterstained with hematoxylin. All sections were examined using a light microscope (Olympus, Tokyo, Japan) and assessed using a JD-801 computer-aided image analyzer. The average absorbance of positive cells in ten randomly selected high-power fields (×400) of each section was used for the analysis of expression.

### Cell culture

H9C2 myoblast cells were maintained in Dulbecco's modified Eagle high glucose medium (DMEM) with 10% fetal bovine serum, 1% penicillin G (100 U/mL), streptomycin (100 mg/mL), and L-glutamine (2 mM)in a humidified atmosphere at 37°C in a CO_2_ incubator. Cell cultures between passages 3 to 5 were used for each experiment.

### Measurement of protein synthesis

[^3^H]Leucine incorporation was measured as described previously [23]. In brief, H9C2 cells were pretreated with different concentrations of quercetin for 30 minutes and subsequently stimulated with Ang II and coincubated with [^3^H]leucine (1 µCi/ml) for 48 h. At the end of the experiment, the cells were washed with phosphate-buffered saline and scraped off the well and then treated with 10% trichloroacetic acid at 4°C for 60 minutes. The precipitates were then dissolved in NaOH (0.1 mol·L^−1^). Aliquots were counted using a scintillation counter. At least three independent sets of experiments were performed.

### Determination of cell surface area

After serum starvation for 24 h, H9C2 cells grown to 60–70% confluency were treated with quercetin (100 µg/ml) and AngII (200 µM) alone or in combination for 48 h in the presence of 1% FBS before size analysis [24]. Images were captured using a bright field microscope (Nikon E600 digital camera attached to an Olympus BX51 microscope) and were then analyzed for cell size using Image Pro Plus 7.0 software (Media Cybernetics).The data shown represent analyses from three independent experiments. The surface area of cells from each group (60 to 84 cells/group) was determined and compared with the control group.

### Immunofluorescence

H9C2 cells were grown on glass coverslips and treated with quercetin (100 µg/ml) and AngII (200 µM) alone or in combination for 48 h in the presence of 1% FBS. Cells were washed twice with PBS, fixed with 4% paraformaldehyde in PBS for 10 minutes and permeabilized with 0.5%Triton X-100 in PBS for 20 minutes. After washing twice with PBS, cells were blocked with 1% FBS and then incubated with anti-PPAR-γ (Abcam UK, dilution 1∶50) or c-fos (Abcam UK, dilution 1∶100), c-jun (Cell Signaling Technology, UK, dilution 1∶200) rabbit polyclonal antibodies overnight at 4°C and then washed twice with PBS. Cells were incubated with FITC-conjugated secondary antibodies for 1 h at room temperature, and the nuclei were counterstained DAPI (0.5 g/mL) with to determine PPAR-γ, c-fos, and c-jun nuclear localization. The coverslips were then washed and mounted on glass slides. Fluorescent images were obtained using appropriate filters with an Olympus BX51 microscope and imaged with an Olympus DP-71 digital camera with an image processing system equipped with Image Pro Plus 7.0 (Media Cybernetics) as described previously [25]–[27].

### PPAR–γ knockdown using an RNAi oligonucleotide

The sequences of the siRNA used to target PPAR-γ (No. NM-001145366) were sense: 5′-CCU CCC UGA UGA AUA AAG ATT-3 ′and antisense: 5′- UCUUUAUUCAUCAGGGAGGTT-3′. A negative control siRNA was used as a control oligonucleotide. The sequences of the negative control siRNA (NCsiRNA) were sense: 5′-UUC UCC GAA CGU GUC ACG UTT-3′, antisense:5′-ACG UGA CAC UGG CGG AGA ATT-3′, and they were synthesized by Gene Pharma (Shanghai, China). Transfections were performed using Lipofectamine 2000 as described previously [28], [29]. Prior to transfection, cells were plated at 4×10^×5^ cells per well in six-well plates in growth medium without antibiotics. The final concentrations of 200 nM PPAR-γ siRNA oligonucleotide was empirically determined to maximally suppress target RNA expression. The siRNA oligonucleotide was transferred to the medium at 48 h prior to treatment with AngII and quercetin. Cells were incubated in Opti-MEM Reduced Serum Medium (Invitrogen) with or without siRNA Lipofectamine 2000 complexes (50 nM) and siRNA Lipofectamine 2000 complexes for 6 h and then replaced with fresh DMEM. After H9C2 cells were transfected with each siRNA for 48 h, cells were incubated with quercetin and AngII alone or in combination for 24 h. Cells were incubated for another 24 h prior to RNA isolation for quantitative real-time PCR analysis and 48 h prior to isolating protein samples for Western blotting analysis.

### RNA isolation and real-time polymerase chain reaction

The mRNA levels of PPAR-γ, c-fos, c-jun, brain natriuretic peptide (BNP), and atrial natriuretic peptide (ANP) were analyzed using RT-PCR. The mRNA sequences were acquired from Gene-bank (NCBI, Bethesda, MD; Table 1). The expression of β-actin was also determined and used as an internal control. Total RNA was isolated from frozen myocardial tissues using TRIzol reagent (Invitrogen, Carlsbad, CA, USA) in accordance with the manufacturer's protocol. RNA quality and concentration were then determined using spectrophotometry (DU 800, Beckman, Palo Alto, CA, USA). Real-time reverse transcriptase-PCR was performed using 1 µg Total RNA for the PrimeScript™ RT reagent Kit with gDNA Eraser (Perfect Real Time; TaKaRa, Kyoto, Japan) and the SYBR Green PCR kit according to the manufacturer's instructions. All real-time polymerase chain reaction experiments were done in trplicate using of cDNA and run for 30 cycles. Relative mRNA expression was determined using the 2^−ΔΔCt^ method [30].

**Table 1:** Primers sequences and real-time PCR amplification parameters.

Gene	Genebank accession no	Forward and reverse primers 5′-3′	Size (bp)	Annealing temperature (°C)
β-actin	NM-031144	CGTTGACATCCGTAAAGACC	142	61
		TAGAGCCACCAATCCACACA		
PPAR-γ	NM-001145366	GCCTGCGGAAGCCCTTTGGT	136	60
		AAGCCTGGGCGGTCTCCACT		
BNP	NM-031545.1	CTTGGGCTGTGACGGGCTGAG	153	58
		GCTGGGGAAAGAAGAGCCGCA		
c-fos	NM-022197.2	CCGCGAACGAGCAGTGACCG	140	61
		AAAGCTCGGCGAGGGGTCCA		
c-jun	NM-021835.3	CGCGGGAGCCAACCAACGTG	141	61
		GCGTCCCCGCTTCAGTAACAAAGT		
ANP	NM-012612.2	TGGGCTCCTTCTCCATCACC	145	60
		GCCAAAAGGCCAGGAAGAGG		

### Western blotting analysis

Protein concentrations were measured using a BCA protein kit to normalize the amount of total protein. Samples containing equal amounts of proteins (30 µg/sample) were separated on a 10% SDS-polyacrylamide gel and then electrophoretically transferred to polyvinylidene fluoride membranes (PVDF). The membranes were washed and blocked in 5% nonfat milk in Tris-buffered saline (TBS). After washing three times in 0.1% Tween 20-TBS (TBST)and then incubating with the appropriate dilution of primary antibodies (PPAR-γ, 1∶500, Abcam Inc., USA; c-fos,1∶1000; c-jun, 1∶1000, Cell Signaling Technology Inc., USA; and anti-β-actin, 1∶2000, Zhongshan Goldenbridge Biotechnology, Beijing, China) at 4°C overnight. They were then incubated with HRP-conjugated secondary antibodies (1∶10000, Bioworld Biotechnology, China) at room temperature for 60 minutes. After incubating with ECL detection reagent, bands were visualized and exposed to X-ray films. The bands were quantified using the ImageJ software (National Institutes of Health, Bethesda, MD). All experiments reported in this study were performed three times. Measurements were expressed as arbitrary units. The results were normalized against β-actin densitometry.

### Statistical analysis

The data are expressed as the mean ± sem and were analyzed using SPSS software (Version 17.0, SPSS Inc., Chicago, IL, USA). For multiple comparisons between groups, one or two-way ANOVAs were used followed by Bonferroni corrected post-hoc tests. Differences were considered to be statistically significant when the P values were less than 0.05. All experiments were repeated at least 3 times.

## Results

### Quercetin decreases systolic blood pressure in spontaneously hypertensive rats

**Figure 2:**
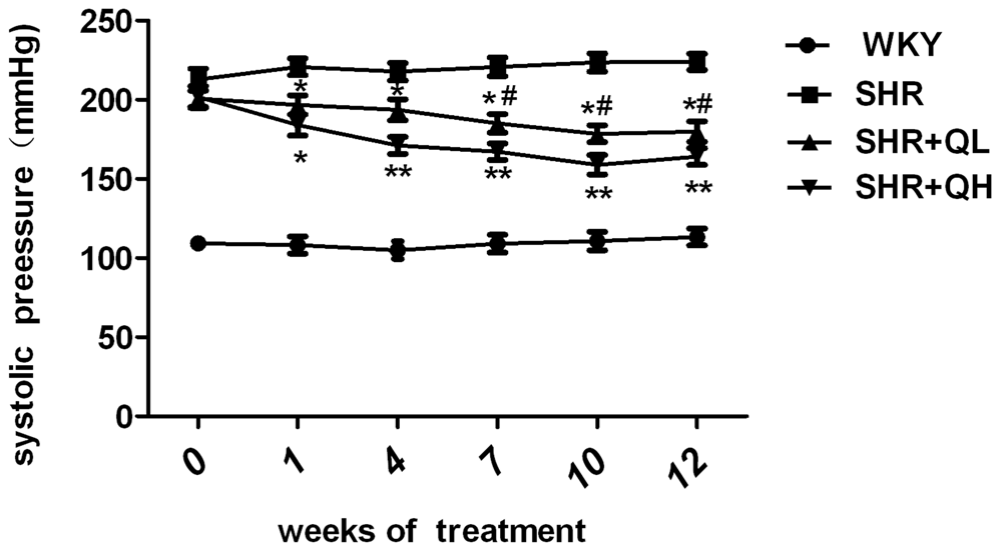
Effects of quercetin on systolic blood pressure measured by tail-cuff plethysmography in the four groups. The characteristics of the systolic blood pressures from week 1 to week 12 in the four groups are shown in Figure 2. Each value represents the mean ± sem (n = 8 in each group). ^*^ *P*<0.05, ^**^ *P*<0.01, vs. the spontaneously hypertensive rats (SHR) group; **^#^** *P*<0.05, vs. the SHR+QH group.

In the week prior to treatment with quercetin, systolic blood pressure in the SHRs was significantly increased compared with the WKY (*P*<0.05). Twenty weeks of quercetin administration induced a significant reduction in SBP in the SHRs, and this effect reached statistical significance after the first week of treatment (*P*<0.05). From the second week of treatment, SBP was significantly decreased in the quercetin-treated groups compared with the SHR group (*P*<0.05), and the decline in the SHR+QH group was distinct from that of the SHR+QL group (*P*<0.05) and was significantly distinct from the 7^th^ to the 12^th^ week (*P*<0.05; Figure 2).

### Cardiac morphology shows that quercetin decreases left ventricular weight index (LVW/BW)

**Figure 3:**
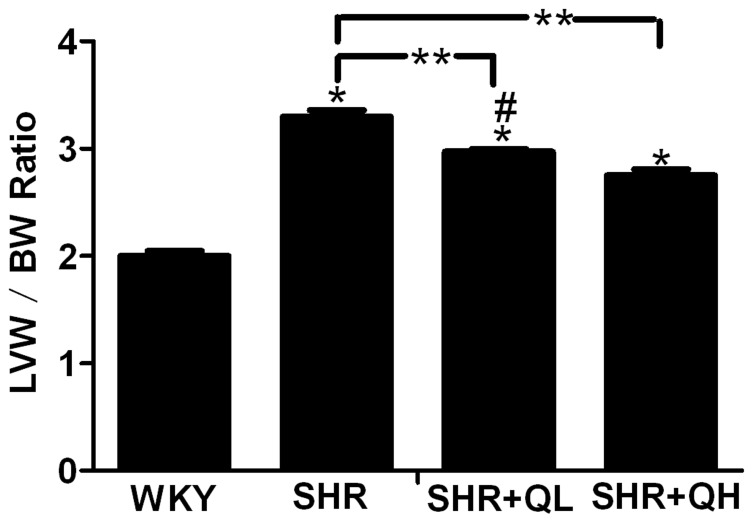
Effects of quercetin on left ventricular weight index (LVW/BW) in the different treatment group. After 12 weeks treatment, the animals were anesthetized and the left ventricular weight (LVW) and body weight (BW) ratio was determined. Each value represents the mean ± sem (n = 8 in each group). ^*^ *P*<0.05, vs. the Wistar–Kyoto rats (WKY) group; ^**^ *P*<0.05, vs. the spontaneously hypertensive rats (SHR) group; **^#^** *P*<0.05, vs. the SHR+QH group.

Neither SHRs or WKY rats showed a change in body weight after treatment with quercetin or vehicle. The left ventricular weight index (LVW/BW) in SHRs was significantly greater than in WKY rats. This parameter was dose-dependently and significantly decreased in quercetin-treated SHRs compared with vehicle-treated SHRs (*P*<0.05; Figure 3).

### Histopathological examination reveals that quercetin attenuates the enlargement in cardiomyocytes and excessive collagen deposition

**Figure 4:**
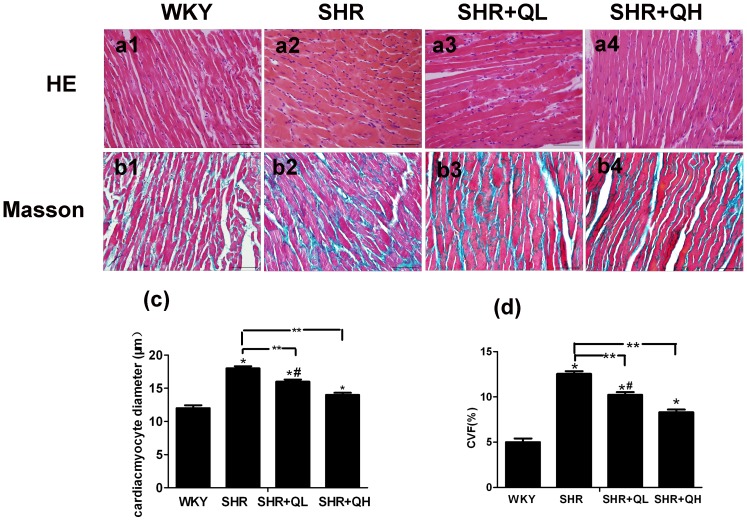
Histopathological analysis reveals that quercetin suppresses myocardial hypertrophy in spontaneously hypertensive rats. Tissue sections were stained with hematoxylin and eosin (H&E; a1–a4; ×400) and Masson's trichrome (b1–b4, ×400). (c) Mean cardiomyocyte diameter was determined by measuring 100 cells from each section. (d) The ratio of the area of myocardial fibrosis (excluding perivascular collagen) to the total myocardial area (percentage) was quantified from images of Masson's trichrome-stained tissue. The scale bar denotes 100 µm. Both mean cardiomyocyte diameter and collagen volume were quantified using Image-Pro Plus software. At least 8–10 areas were evaluated in each section and each value denotes the mean ± sem (n = 8 in each group). CVF, collagen volume fraction. ^*^ *P*<0.05, vs. the Wistar–Kyoto rats (WKY) group; ^**^ *P*<0.05, vs. the spontaneously hypertensive rats (SHR) group; **^#^** *P*<0.05, vs. the SHR+QH group.

Pathological changes were significantly attenuated by quercetin. Hematoxylin and eosin staining was performed for all groups (Figure 4.a1–a4). Compared with the WKY group, cardiac muscle fibers were enlarged and disorganized in the SHR group. Hematoxylin and eosin-stained myocardial tissue in the quercetin-treated SHR groups showed reductions in the size of the cardiomyocytes and reduced fibrosis. In addition, cardiac muscle fibers were also better-arranged. Masson's trichrome staining was performed to assess the effect of quercetin on cardiac fibrosis (Figure 4.b1–b4). The WKY myocardium showed a normal array of myocardial fibers and very little interstitial collagen. Quercetin administration dramatically and dose-dependently attenuated the enlargement in myocyte size and the increase in collagen volume fraction compared with SHRs (*P*<0.05; Figure 4.c–d).

### Transmission electron microscopy shows that quercetin attenuates SHRs ultrastructural pathology

**Figure 5:**
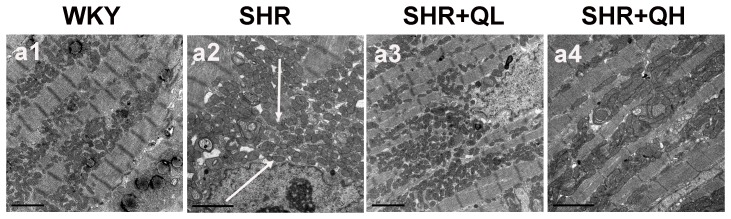
Transmission electron microscopic observations of myocardial tissue of rats in the different treatment group. Ultrastructure of cardiac sections (a1–a4; ×8000). (a1) Observations of myocardial tissue of the WKY vehicle-treated group, which showed normal myocardial tissue. (a2) Myocardial tissue in the SHR group. Swollen mitochondria, deposition of a large number of dense granules in the mitochondria and cellular necrosis was observed. (a3, a4) Myocardial tissue of the quercetin-treated SHR groups. The cardiomyocyte mitochondria were less swollen, the number of dense granules was lower and the structure was relatively better organized compared with the SHR group. Arrows indicate obviously swollen and loosely arranged mitochondria.

In the WKY group, arrays of myofibrils were closely arranged in an orderly manner within the sarcomere, and mitochondria were of normal size and present in normal numbers. However, in the SHR group, the mitochondrial structure was damaged severely and cellular or tissue swelling was apparent. Most myofibrils had either disappeared or were poorly arranged. Mitochondria were noticeably swollen and loosely arranged. In addition, mitochondrial membranes were vague or partly ruptured and cristae were clearly loose or dissolved with many vacuoles. The significant change in ultrastructural organization of mitochondria and myofibrils was attenuated in the quercetin-treated SHR groups with more obvious improvements noted in the high-dose group (Figure 5. a1–a4).

### Echcardiography shows that quercetin improves cardiac function in spontaneously hypertensive rats

**Figure 6:**
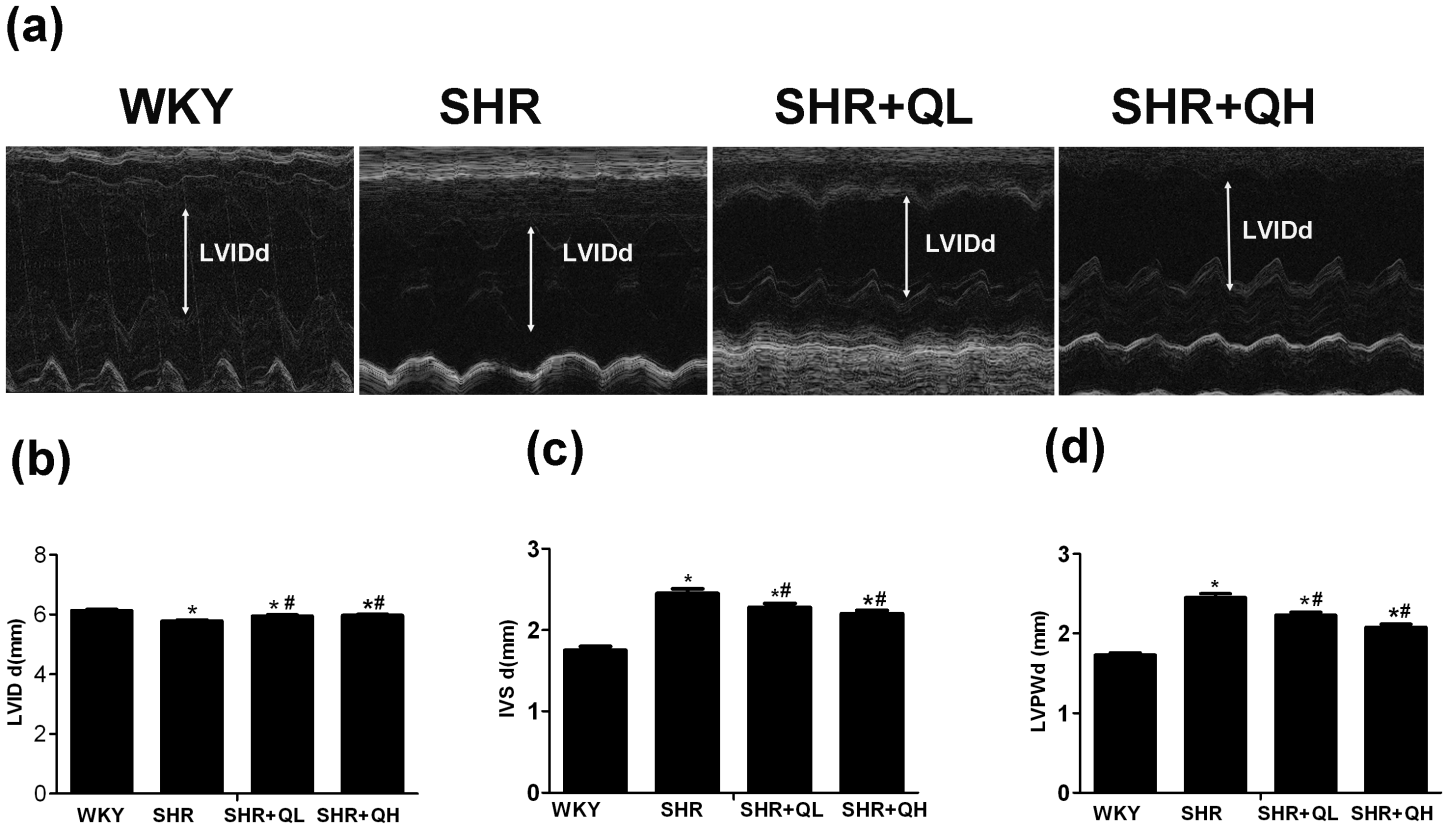
Quercetin administration improves cardiac function in spontaneously hypertensive rats. Representative M-mode echocardiogram showing improved wall motion after treatment (a–d). After 12 weeks of treatment with quercetin, the left ventricular end-diastolic internal diameter (LVIDd) improved and the left ventricular end-diastolic posterior wall thickness (LVPWd), and end-diastolic interventricular septal thickness (IVSd) differences were attenuated. Data are the mean ± sem (n = 8 for each group). ^*^ *P*<0.05, vs. the Wistar–Kyoto rats (WKY) group ; **^#^** *P*<0.05, vs. the spontaneously hypertensive rats (SHR) group.

Cardiac performance was analyzed using echo-cardiography to investigate cardiac function in vivo after quercetin administration (Figure 6; Table 2). Compared with the WKY group, LVPWd and IVSd were both significantly increased at week 1 or 12 of the experiment but LVIDd decreased by the 12^th^ week in the SHR group (*P*<0.05; Table 2). Following 12 weeks of quercetin treatment, LVIDd was significantly increased but IVSd and LVPWd were obviously decreased compared with the SHRs (*P*<0.05). Moreover, the mid-wall fractional shortening and E/A decreased significantly in SHRs and were significantly improved at week 12 in SHRs treated with quercetin (*P*<0.05; Table 2), which indicates that quercetin mitigated left ventricular hypertrophy and improved cardiac function.

**Table 2:** Echocardiographic data from the four treatment groups at week 1 and week12.

	Week 1				Week 12			
	WKY	SHR	SHR+QL	SHR+QH	WKY	SHR	SHR+QL	SHR+QH
LVIDd(mm)	5.27±0.04	5.30±0.03	5.28±0.04	5.31±0.03	6.14±0.04	5.79±0.03*	5.95±0.04* #	5.97±0.05* #
IVSd (mm)	1.32±0.03	1.71±0.03*	1.70±0.04*	1.72±0.03*	1.75±0.05	2.45±0.06*	2.28±0.05* #	2.20±0.04* #
LVPWd(mm)	1.32±0.04	1.68±0.03*	1.69±0.05*	1.70±0.03*	1.73±0.04	2.45±0.05*	2.23±0.04* #	2.08±0.04* #
LVEF(%)	80.21±0.26	79.78±0.14	79.78±0.24	78.67±0.15	79.88±0.22	79.80±0.16	79.80±0.14	79.78±0.24
E/A	2.02±0.03	1.98±0.02	1.98±0.03	1.99±0.02	2.11±0.02	1.85±0.02*	1.96±0.03* #	1.98±0.03* #
MFS (%)	24.74±0.30	24.72±0.24	24.77±0.23	24.76±0.22	24.88±0.33	22.33±0.24*	23.42±0.23* #	23.82±0.24* #

Abbreviations: LVIDd, left ventricular end-diastolic internal diameter; IVSd, end-diastolic interventricular septal thickness; LVEF, left ventricular ejection fraction; LVPWd, left ventricular end-diastolic posterior wall thickness; E/A, ratio of the early to the late peak diastolic transmitral flow velocity; MFS, midwall fractional shortening; SHR, spontaneously hypertensive rat; WKY, Wistar–Kyoto rat; SHR+QH, high-dose quercetin-treated group; SHR+QL, low-dose quercetin-treated group. Data are expressed as the mean ± sem (n = 8 for each group).

* *P*<0.05, vs. the WKY group;

# *P*<0.05, vs. the SHR group.

### Immunohistochemical staining shows that quercetin increases PPAR-γ and suppresses AP-1 protein expression

**Figure 7:**
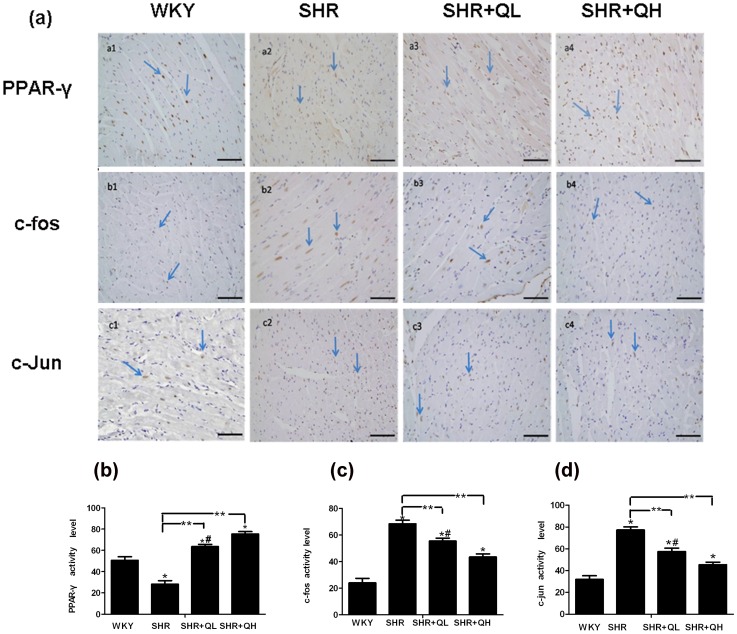
Quercetin administration activates protein expression of PPAR-γ and inhibits protein expression of AP-1 in cardiomyocyte. Figure (a2) shows few cells with nuclei positive for PPAR-γ in spontaneously hypertensive rat cardiomyocytes. However, SHRs (a3–a4) treated with quercetin showed significantly more positive nuclear staining that followed a dose-dependent manner. Compared with Wistar–Kyoto (WKY) rats (b1, c1), SHRs (b2, c2) treated with vehicle showed a large number of positively stained cardiomyocyte nuclei. In contrast, SHRs treated with quercetin (b3–b4, c3–c4) had significantly and dose-dependently fewer positively stained cardiomyocyte nuclei. The scale bar denotes 100 µm (×400). (b–d) At least 8–10 areas were evaluated in each section and were quantified using Image-Pro Plus software. Arrows indicate positive nuclear staining. Results are shown as the mean ± sem. ^*^ *P*<0.05, vs. the Wistar–Kyoto rats (WKY) group; ^**^ *P*<0.05, vs. the spontaneously hypertensive rats (SHR) group; **^#^** *P*<0.05, vs. the SHR+QH group.

The protein expression of PPAR-γ and AP-1(c-fos, c-jun) was examined in the myocardial tissue. Compared with the WKY group, AP-1(c-fos, c-jun) was significantly increased after 12 weeks of treatment in cardiomyocytes of the SHR group (*P*<0.05). The level of PPAR-γ expression was markedly and dose-dependently increased in the quercetin-treated SHR groups compared with the SHR group (*P*<0.05). However, AP-1 (c-fos, c-jun) activation was drastically and dose-dependently suppressed in the quercetin-treated groups compared with the SHR group (*P*<0.05; Figure 7. a–d).

### Real-time polymerase chain reactions show that quercetin increases PPAR-γ mRNA and suppresses AP-1mRNA expression

**Figure 8:**
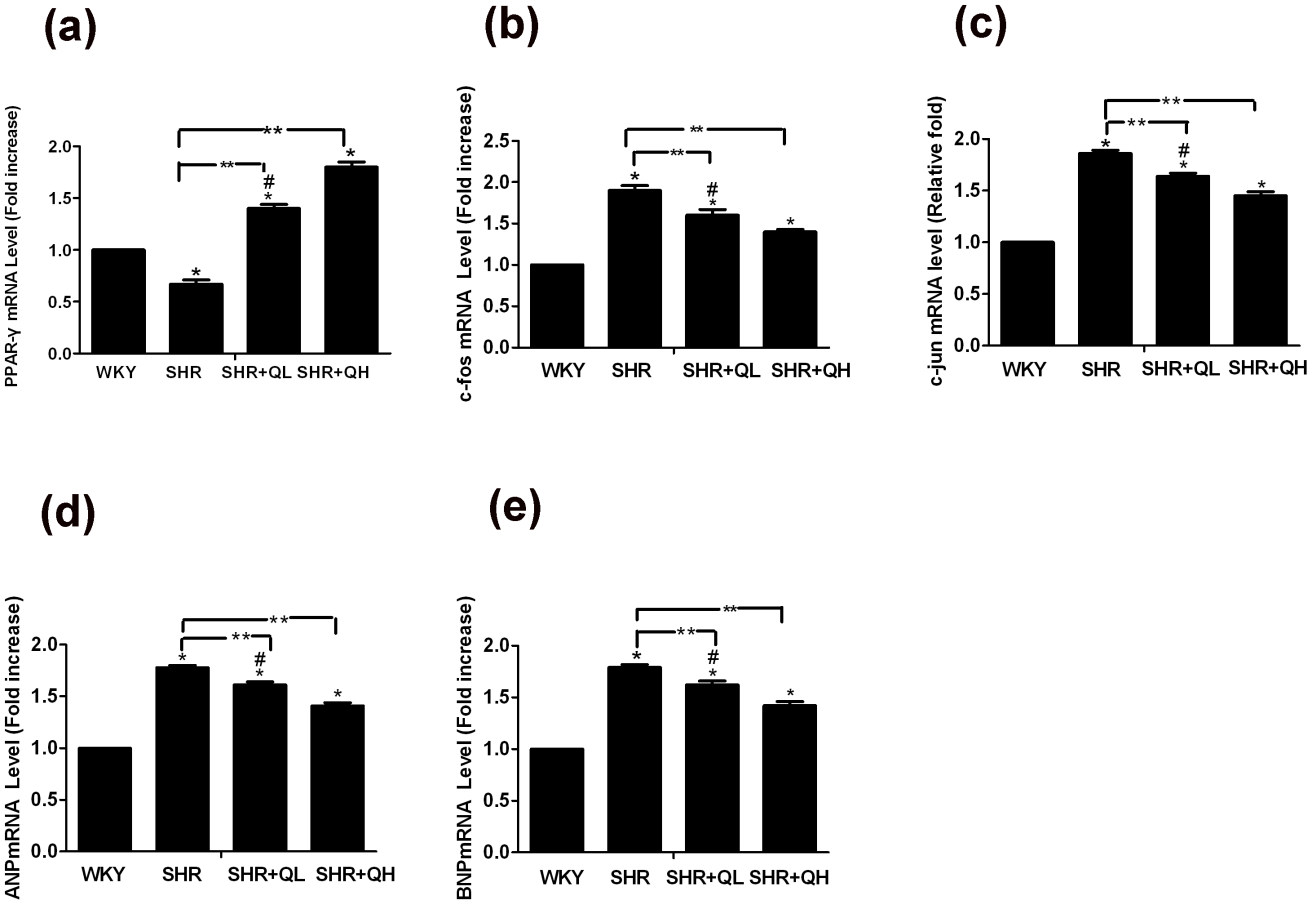
Quercetin enhances PPAR-γ mRNA expression and suppresses AP-1 mRNA and BNP and ANP mRNA levels after treatment. After 12 weeks of treatment with quercetin, the myocardial tissues mRNA level were analysis by real-time polymerase chain reaction. Values denote the expression level relative to the Wistar–Kyoto (WKY) group (n = 8 per group). All samples were analyzed in triplicate and are expressed as the mean ± sem. ^*^ *P*<0.05, vs. the Wistar–Kyoto rats (WKY) group; ^**^ *P*<0.05, vs. the spontaneously hypertensive rats (SHR) group; ^#^ *P*<0.05, vs. the SHR+QH group.

We examined mRNA expression levels of PPAR-γ, AP-1 (related to hypertrophy), ANP and BNP (markers of hypertrophy) in each group using real-time PCR. The results showed that all tissues expressed these mRNAs constitutively but that the expression levels varied between these groups. The expression of PPAR-γ mRNA in the quercetin-treated groups was significantly higher than in the SHR group in a dose dependent manner (*P*<0.05). In contrast, the expressions of c-fos mRNA, c-jun mRNA, ANP mRNA and BNP mRNA were elevated in the SHR group but reduced in the quercetin-treated groups in a dose-dependent manner (*P*<0.05; Figure 8. a–e).

### Western blotting reveals that quercetin increases PPAR-γ protein and suppresses AP-1 protein expression

**Figure 9:**
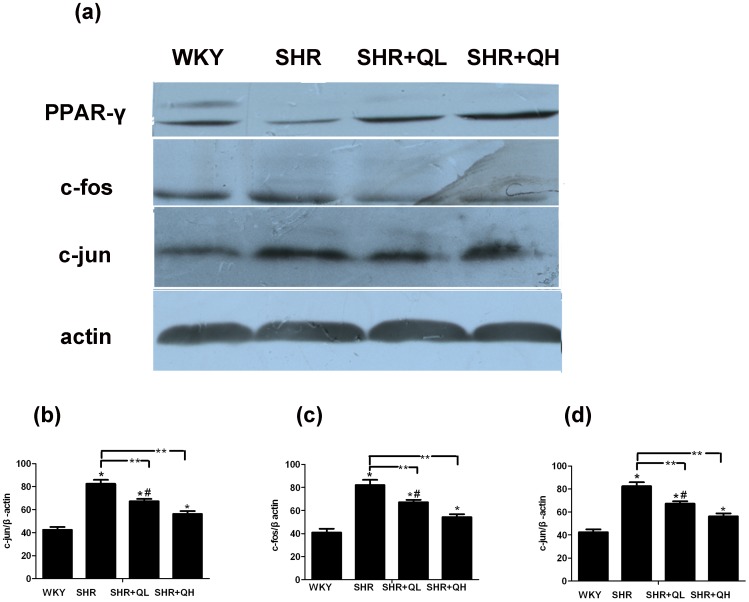
Western blotting reveals that quercetin enhances PPAR-γ protein expression and suppresses AP-1 protein expression. After 12 weeks of treatment with quercetin, the myocardial tissues protein expression were analysis by Western blotting. All samples were analyzed in triplicate and expressed as the mean ± sem. ^*^ *P*<0.05, vs. the Wistar–Kyoto rats (WKY) group; ^**^ *P*<0.05, vs. the spontaneously hypertensive rats (SHR) group; ^#^ *P*<0.05, vs. the SHR+QH group.

PPAR-γ and AP-1(c-fos, c-jun) protein levels were determined by analyzing bands from western blotting bands using image software. The expression levels of these three proteins in myocardial tissue were normalized to β-actin. Following 12 weeks of treatment with quercetin, the expression of PPAR-γ was significantly enhanced in a dose dependent manner compared with the SHR group (*P*<0.05). However, AP-1(c-fos and c-jun) were attenuated in a dose-dependent manner following 12 weeks of treatment with quercetin compared with the SHR group (*P*<0.05; Figure 9. a–d).

### Quercetin ininhibits AngII-induced protein synthesis in H9C2cells

**Figure 10:**
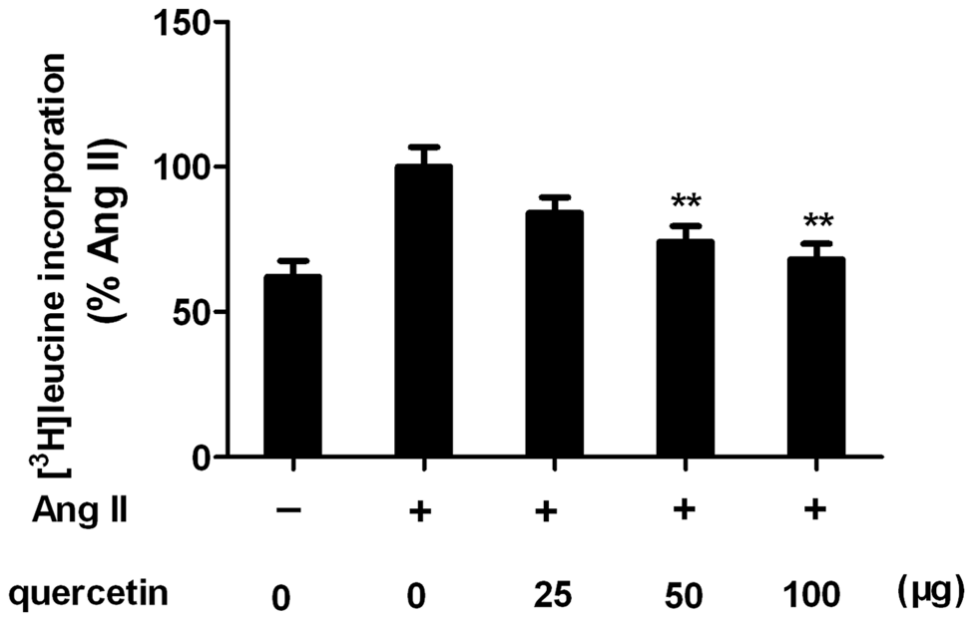
Inhibitory effects of quercetin on Ang II–stimulated [^3^H]leucine (1 µCi/ml) incorporation into H9C2 cells. H9C2 cells were pretreated with different concentrations of quercetin for 30[^3^H]leucine(1 µCi/ml) for 48 h. All values were expressed as a percentage of incorporation in the comparison to Ang II treated group. Data are mean ± sem and each experiment performed in triplicate independently. ^**^ *P*<0.01, vs Ang II-treated group.

The above experimental results indicate that quercetin may attenuate cardiac hypertrophy in vivo. To test this hypothesis, we determined the effect of inhibition of quercetin on AngII induced protein synthesis in vitro. As expected, Ang II strongly induced [^3^H]leucine incorporation pretreated cells. However, in quercetin pretreated cells, Ang II-mediated incorporation was markedly reduced in a concentration-dependent manner (Figure 10). At 100 µg/ml, protein synthesis was reduced to almost basal levels (64% reduction of Ang II stimulated [^3^H]leucine incorporation).

### Improvement of AngII-induced hypertrophy by quercetin in H9C2 cells

**Figure 11:**
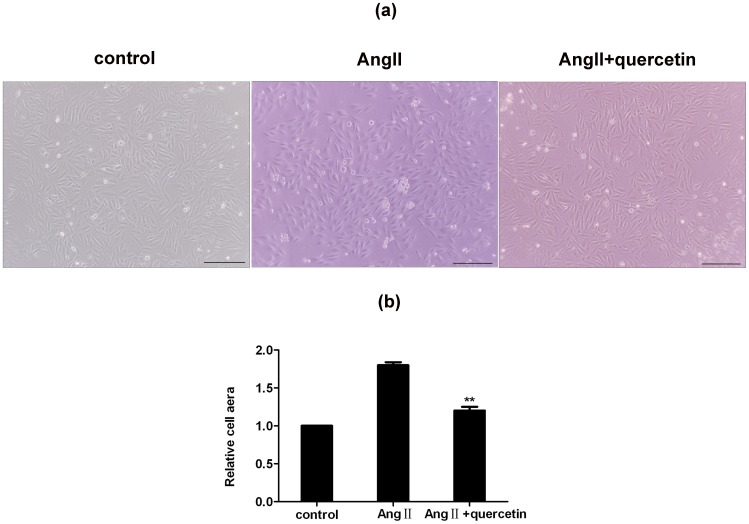
AngII induced hypertrophic growth of H9C2 cells was inhibited by quercetin. After treated with quercetin (100 µg/ml) and AngII (200 µM) alone or in combination for 48 h (a), H9C2 cells hypertrophy was analyzed by measuring cell surface area using Image Pro Plus software 7.0. (b). Ang II (200 µM) caused an increase in H9C2 cells size and was attenuated by quercetin (100 µg/mL). The values denote the relative area ± sem (n = 3). ^**^ *P*<0.01, vs. the Ang II-treated group.

To illustrate the effect of quercetin on hypertrophic H9C2 cells, we explored the changes of one key parameter of hypertrophic myocytes (the surface area). We used 100 µg/ml quercetin in our experiments to study its mechanism of action because this concentration showed maximal inhibition on AngII-induced H9C2 cells hypertrophy and was also nontoxic to H9C2 cells when they were treated for 24 h at this concentration. After serum starvation for 24 h, H9C2 cells were treated with quercetin (100 µg/ml) and Ang II (200 µM) alone or in combination for 24 h with 1% FBS before analyzing size (Figure 11. a). Ang II stimulation significantly increased the surface area of H9C2 cells (1.8-fold vs control group, n = 200 cells, *P*<0.01). Quercetin (100 µg/mL) pretreatment significantly attenuated this increase in H9C2 cells compared with the Ang II group (0.65-fold, n = 200 cells, *P*<0.01; Figure 11. b).

### Immunofluorescence shows that quercetin increases PPAR-γ protein and suppresses AP-1 protein expression in H9C2 cells

**Figure 12:**
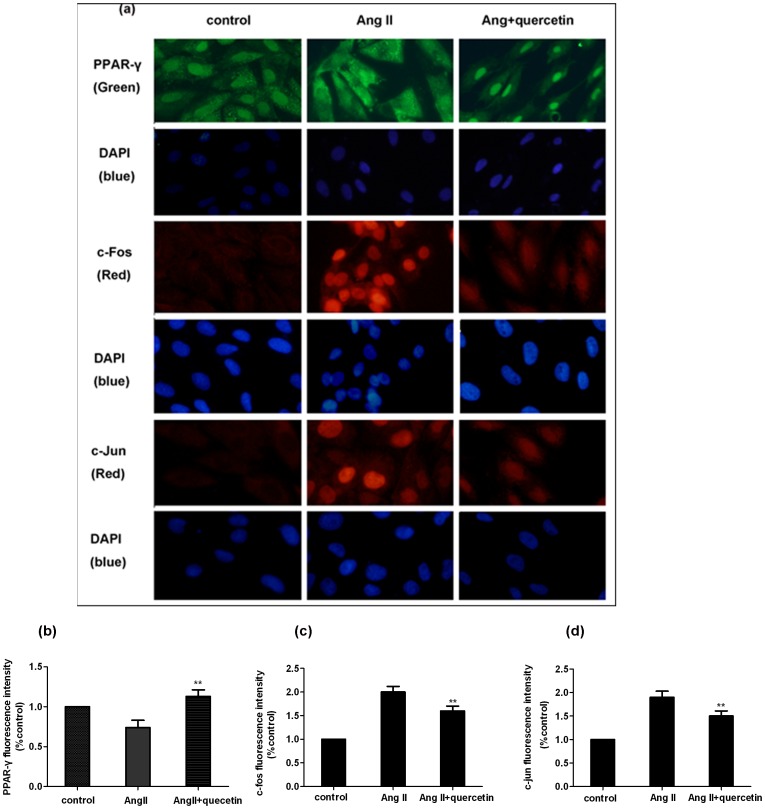
Cells treated with quercetin enhances PPAR-γ nuclear immunofluorescence and suppresses AP-1 nuclear immunofluorescence activity. After H9C2 cells were treated with quercetin (100 µg/ml) and AngII (200 µM) or in combination for 24 hours, (a) Coverslips were mounted on a slide and processed for fluorescence (×400), then analyzed using Image Pro Plus 7.0. Green: PPAR-γ; Red: c-fos and c-jun; Blue: nuclei (DAPI). Results are representative images of cells from three independent experiments. (b–d) The protein levels of PPAR-γ, c-fos and c-jun were quantified by densitometry, and the data denote the relative intensity compared with control cells. The values denote the relative area ± sem (n = 3). ^**^ *P*<0.01, vs. the Ang II-treated group.

The fluorescence intensity of nuclear PPAR-γ was lower whereas AP-1 (c-fos, c-jun) expression was increased in the AngII-treated group compared with the control group (*P*<0.01; Figure 12.a). However, if H9C2 cells were treated with quercetin (100 µg/ml) and AngII (200 µM) or in combination for 24 hours, the fluorescence of PPAR-γ was significantly increased (*P*<0.01; vs AngII group). In parallel, the fluorescence of c-fos and c-jun was significantly decreased in the quercetin and AngII combination treatment group compared with the Ang II group (*P*<0.01; Figure 12.b–d).

### Western blotting shows that PPAR-γ is involved in the inhibitory effects of quercetin on Ang II-induced AP-1 activation

**Figure 13:**
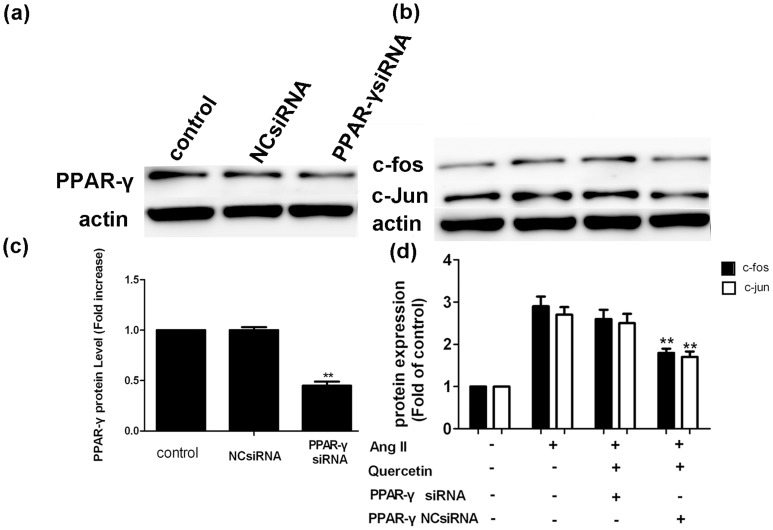
Cells transfected with PPAR-γ siRNA showed a reversal of the quercetin inhibition on AP-1 protein expression. H9C2 cells were transiently transfected with siRNA targeted to PPAR-γ and this siRNA led to decrease in protein levels of PPAR-γ compared with NCsiRNA and untreated controls (*P*<0.01; a, c). After H9C2 cells were transfected with PPAR-γ NCsiRNA or PPAR-γ siRNA (200 nM) for 24 h, cells were incubated with quercetin and Ang II alone or in combination for 24 h. Cells were incubated for a further 48 h prior to isolating protein samples for c-fos and c-jun western blotting analysis (b, d). All samples were analyzed in triplicate, and data are expressed as the mean ± sem. ^**^ *P*<0.01, vs. Ang II-treated group.

The experimental results above suggested that activation of the PPAR-γ pathway may be involved in the quercetin in inhibition of Ang II-induced hypertrophic growth of H9C2 cells. To test this hypothesis, PPAR-γ siRNA was used to evaluate the possible involvement of PPAR-γ in the quercetin-mediated inhibition of Ang II-induced H9C2 cells hypertrophy. H9C2 cells were transiently transfected with siRNA targeted to PPAR-γ and a decrease in protein levels of PPAR-γ was observed compared with NCsiRNA and untreated controls (*P*<0.01; Figure 13. a, c.). However, the elevated expression of AP-1 (c-fos, c-jun) in H9C2 cells induced by AngII was not inhibited by treatment with quercetin after cells were transfected with the PPAR-γ siRNA (Figure 13. b, d). These results suggest that the effect of quercetin on AP-1(c-fos, c-jun) expression in Ang II-induced H9C2 cells hypertrophy was, at least in part, via a PPAR-γ-dependent pathway.

### PPAR-γ is involved in the inhibitory effects of quercetin on Ang II-induced ANP and BNP mRNA

**Figure 14:**
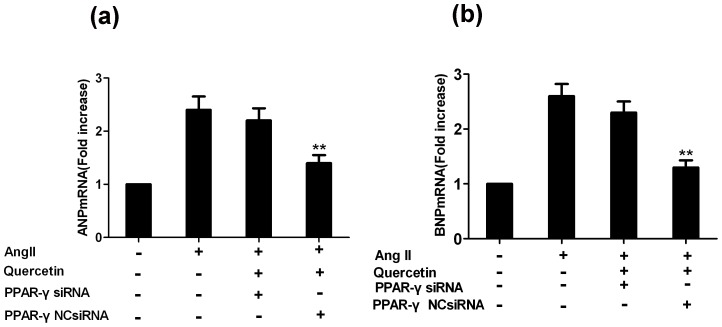
Transfection with PPAR-γ siRNA reversed the quercetin-mediated inhibition of ANP and BNP mRNA expression. After H9C2 cells were transfected with each siRNA (200 nM) for 24 h, cells were treated with quercetin and AngII alone or in combination for 24 h. Cells were incubated for a further 24 h before RNA isolation for analysis of ANP and BNP mRNA by real-time PCR (a–b). Data are expressed as the mean ± sem. Values are the means from three independent experiments. ^**^ *P*<0.01, vs. Ang II-treated group.

To establish whether PPAR-γ is critically involved in the transcriptional regulation of the ANP and BNP genes (markers of cardiac hypertrophy) after treatment with quercetin, cells were transiently transfected with siRNA targeted to PPAR-γ. Transfection with PPAR-γ siRNA increased ANP and BNP compared with the control group (*P*<0.01; Figure 14. a–b) indicating that PPAR-γ may play an important role in controlling the transcription of ANP and BNP in quercetin-mediated inhibition of Ang II-induced hypertrophic growth of H9C2 cells.

## Discussion

Cardiac hypertrophy is a pathological process that occurs in response to an increase in blood pressure during the development of various cardiac diseases [31]. In epidemiological studies, increasing dietary intake of flavonoids is accompanied by a reduced risk of cardiovascular disease. Quercetin has become the subject of widespread attention primarily because of its broad spectrum of putatively beneficial effects on the cardiovascular system.

The flavonoid quercetin is one of the most abundant polyphenolic compounds and is found in many fruits and vegetables. Various pharmacological studies have confirmed that quercetin reduces elevated blood pressure and ameliorates endothelial dysfunction and end-organ injury (cardiac, renal hypertrophy) in the spontaneously hypertensive rats [15]. Furthermore, quercetin has been demonstrated to prevent VSMC during Ang II-induced hypertrophy by inhibiting the JNK and AP-1 signaling pathway during arteriosclerosis [32]. The peroxisome proliferator-activated receptor (PPAR) γ is a ligand-activated transcription factor that belongs to the nuclear receptor superfamily. In recent years, great numbers of effects of PPAR-γ have been reported in the regulation of myocardial cells, endothelial cells, vascular smooth muscle cells, and macrophages in myocardial tissue [33]–[37]. It has also been identified in the heart, and previous studies have shown that it plays a pivotal role during all stages of the cardiac hypertrophy process of various cardiac diseases [14], [38].

We hypothesized that quercetin-mediated inhibition of cardiac hypertrophy would be accompanied by increased activation of PPAR-γ and reduced activation of AP-1 (c-fos, c-jun) both in vivo and in vitro. Quercetin administration significantly reduced the systolic blood pressure and LVW/BW ratio in vivo in a dose-dependent manner. Treatment with quercetin also markedly decreased myocyte size or collagen I and III expression upon histopathological examination and significantly decreased IVSd and LVPWd as assessed by echocardiography compared with SHRs. Transmission electron microscopy showed few deleterious changes in myocardial tissue of quercetin-treated SHRs compared with the SHR control group. In vitro, results also demonstrated that quercetin suppressed Ang II-induced H9C2 cells hypertrophy including H9C2 cells surface area and protein synthesis. In parallel, we found that PPAR-γ activation may play a key role in the cardioprotection of quercetin (the inhibition of cardiac hypertrophy). The results showed that the levels of PPAR-γ were markedly increased in the quercetin-treated groups when analyzed using immunofluorescence or immunohistochemical assays. Conversely, AP-1(c-fos,s-jun) activation was suppressed in these groups and decreases were also observed in the downstream hypertrophy genes including the in vivo levels of ANP and BNP (*P*<0.05,vs. SHRs). Additionally, both western blotting and real time-PCR demonstrated that PPAR-γ protein and mRNA in the myocardium was increased whereas AP-1(c-fos, s-jun) protein and mRNA was significantly decreased in the quercetin-treated groups (*P*<0.05, vs. SHRs). Western blotting showed that siRNA-mediated inhibition of PPAR-γ transcription led to an increase in AP-1(c-fos, c-jun) protein expression in H9C2 cells, indicating that quercetin, at least in part, deregulates c-fos and c-jun via the PPAR-γ signaling pathway. In real time-PCR experiments, we found that the PPAR-γ-targeted siRNA resulted in an increase in ANP and BNP expression. The expression of ANP and BNP was abolished in cells transfected with NC siRNA compared with the PPAR-γ-targeted siRNA group. Therefore, we propose that the PPAR-γ effect may play a crucial role in the prevention of cardiac hypertrophy. The inhibitory effects of quercetin on AP-1 lead directly to a decrease in expression of ANP and BNP hypertrophic genes.

In conclusion, we suggest that quercetin may ameliorate cardiac hypertrophy by activating the PPAR-γ signaling pathway. This study is the first to demonstrate that quercetin effectively prevented cardiac hypertrophy by suppressing AP-1(c-fos,c-jun) transcription activity, which may help in further inhibiting the transcription of downstream genes that are involved in cardiac hypertrophy. Therefore, it is conceivable that quercetin attenuates cardiac hypertrophy by enhancing PPAR-γ expression and suppressing AP-1 (c-fos, c-jun) activity, thereby causing a decrease in the transcription of the downstream hypertrophy genes BNP and ANP. The findings of this study may shed light on a potential mechanism underlying quercetin-mediated attenuation of cardiac hypertrophy.
